# ﻿*Astragalus zibaishanensis* sp. nov. (Fabaceae), a new species from Shaanxi, China

**DOI:** 10.3897/phytokeys.266.170977

**Published:** 2025-11-19

**Authors:** Pei-Liang Liu, Lu-Lu Xun, Chun Su, Yuan Lu, Bin Li, Ming Yue

**Affiliations:** 1 Key Laboratory of Resource Biology and Biotechnology in Western China, Ministry of Education, Northwest University, Xi’an, Shaanxi, China Northwest University Xi'an China; 2 Shaanxi Engineering Research Centre for Conservation and Utilization of Botanical Resources, Xi’an Botanical Garden of Shaanxi Province (Institute of Botany of Shaanxi Province), Xi’an, Shaanxi, China Shaanxi Engineering Research Centre for Conservation and Utilization of Botanical Resources, Xi’an Botanical Garden of Shaanxi Province (Institute of Botany of Shaanxi Province) Xi'an China

**Keywords:** Fabaceae, new species, phylogeny, taxonomy

## Abstract

*Astragalus zibaishanensis***sp. nov.** (Fabaceae) is described and illustrated from Shaanxi, China. This new species is similar to *Astragalus monadelphus*, *A. xitaibaicus* and *A. neomonodelphus* by having yellow petals, pseudomonadelphous androecium, and stipitate legumes, but can be distinguished by its stem 0.9–1.5 mm in diameter; leaflets 5–7, hairy adaxially and abaxially; calyx teeth triangular, ca. 0.5 mm long; standard 17–20 mm long; wings 18–21 mm long; keels 17–20 mm long; pistil glabrous; legume glabrous, with a stipe 10–11 mm long. *Astragalus zibaishanensis* is unique in *Astragalus* by having pseudomonadelphous androecium. Phylogenetic analyses confirm that the new species is a member of A. sect. Cenantrum. The nuclear ITS tree shows that *A. zibaishanensis* forms a clade with *A. henryi*, *A. umbellatus*, *A. chilienshanensis*, *A. moellendorffii*, *A. przewalskii*, *A. monadelphus*, *A. xitaibaicus* and *A. neomonodelphus*.

## ﻿Introduction

*Astragalus* L. (Fabaceae) is the largest plant genus, and more than 3200 species have been recognized worldwide (Moonlight et al. 2024). The genus is distributed in various habitats in temperate northern hemisphere, with less species in the southern hemisphere. The genus exhibits outstanding species richness in arid and semi-arid areas in Asia and North America with great ecological importance (Folk et al. 2024). Molecular phylogenetic analyses have revealed intergeneric and intrageneric relationships of *Astragalus* (Wojciechowski et al. 1999; Kazempour Osaloo et al. 2003; Wojciechowski 2005; Duan et al. 2024; Folk et al. 2024), and 11 intrageneric clades have been recognized (Azani et al. 2017, 2019; Su et al. 2021a; Buono et al. 2025). Within *Astragalus*, the clade *Phaca* is an early-divergent group which includes perennial herbaceous species with basifixed hairs, large yellow petals, mostly bilocular or semi-bilocular legumes (Azani et al. 2017, 2019; Su et al. 2021a; Folk et al. 2024). Within the *Phaca* clade, Astragalus sect. Cenantrum Bunge is generally characterized by well-developed stems, free stipules, imparipinnate leaves, peduncled racemes, campanulate calyx and stipitate unilocular papery legumes (Podlech and Zarre 2013). In this section, there are 41 species in Eurasia (Podlech and Zarre 2013), and one species, *A. americanus* (Hook.) M. E. Jones, is endemic to North America (Isely 1998).

About 430 species of *Astragalus* have been documented from China (Chai and Yan 2010; Podlech 2010; Xu and Podlech 2010; Liu et al. 2011; Zhao 2012; Li and Yu 2014; Wu 2015; Ri and Zhao 2018; Liu et al. 2020; Hu et al. 2021; Su et al. 2021b; Zhang et al. 2021; Xu et al. 2023; Ke et al. 2024; Yang et al. 2024; Wu 2025; Yang et al. 2025). A total of 28 species of Astragalus sect. Cenantrum are known from China (Podlech 2010; Xu and Podlech 2010; Podlech and Zarre 2013). In the fieldwork in Mt. Zibaishan, Shaanxi, China, we found a species belonging to A. sect. Cenantrum, which was revealed as a new species by detailed morphological comparison and phylogenetic analyses.

## ﻿Material and methods

### ﻿Taxon sampling

Three accessions of the new species were sampled from the field. Because the new species is morphologically similar to *A. monadelphus* Bunge ex Maxim., *A. xitaibaicus* (K. T. Fu) Podlech & L. R. Xu and *A. neomonodelphus* H. T. Tsai & T. T. Yu, these three species were also sampled for phylogenetic analyses. We also sampled another 15 species in A. sect. Cenantrum to test the phylogenetic position of the new species in this section. Previous phylogenetic analysis showed that species of A. sect. Skythropos N. D. Simpson and A. sect. Ebracteolati N. D. Simpson were nested in A. sect. Cenantrum (Su et al. 2021a), therefore *A. skythropos* Bunge and *A. craibianus* N. D. Simpson were also sampled. Representative species of other clades of *Astragalus* (Su et al. 2021a) were included in phylogeny. *Oxytropis bicolor* Bunge was selected as the outgroup. Sample information was shown in Appendix 1.

### ﻿DNA extraction, PCR and sequencing

Genomic DNA was extracted from silica-gel dried leaves by using the Qiagen DNeasy® Plant Mini Kit (Hilden, Germany). The nuclear ribosomal internal transcribed spacer (ITS), and the plastid *psbA-trnH* and *trnL-F* sequences were amplified by Polymerase chain reactions (PCR). Primers were ITS5 and ITS4 (White et al. 1990) for ITS, psbAF and trnHR (Sang et al. 1997) for *psbA-trnH*, c and f (Taberlet et al. 1991) for *trnL-F* sequences. PCR conditions followed Liu et al. (2017). Amplicons were sequenced in both directions by using the PCR primers. Sequences were assembled in Geneious Prime® 2025.0.2 (https://www.geneious.com). Newly generated sequences have been deposited in the GenBase (Bu et al. 2024) in National Genomics Data Center (CNCB-NGDC Members and Partners 2025), Beijing Institute of Genomics, Chinese Academy of Sciences/China National Center for Bioinformation. The GenBase accession numbers were listed in Appendix 1, and the data were publicly accessible at https://ngdc.cncb.ac.cn/genbase.

### ﻿Phylogenetic analysis

Phylogenetic analyses were conducted based on the newly generated sequences together with the data from Su et al. (2021a) and several sequences from GenBank (Appendix 1). Multiple sequence alignments were performed in Geneious Prime®. The best-fit nucleotide substitution model for each alignment was determined by using jModelTest v.2.1.7 with the Bayesian Information Criterion (BIC) (Darriba et al. 2012). The models for the ITS, *psbA-trnH* and *trnL-F* sequences were K80+G, F81+I+G and F81+G, respectively. The nuclear and the plastid data were analyzed separately. The plastid *psbA-trnH* and *trnL-F* sequences were concatenated and partitioned in phylogenetic reconstructions by using respective models. MrBayes v.3.2.5 (Ronquist and Huelsenbeck 2003; Ronquist et al. 2012) was used to conduct Bayesian inference (BI). BI was run for 10,000,000 generations, and trees were sampled every 1,000 generations. The first 2,500 trees were discarded, and the remaining 7,500 trees were used to build a 50% majority-rule consensus tree and posterior probabilities (PP). The maximum parsimony (MP) and maximum likelihood (ML) analyses were conducted by using PAUP* 4.0a169 (Swofford 2002) and RAxML v.8.2 (Stamatakis 2014), respectively. The MP and ML bootstrap analyses were performed with 1,000 replicates. Bootstrap support percentages (BSMP, BSML) from the MP and ML analyses were labeled on the corresponding branches of the BI trees.

## ﻿Results

### ﻿Nuclear data

**Figure 1.**
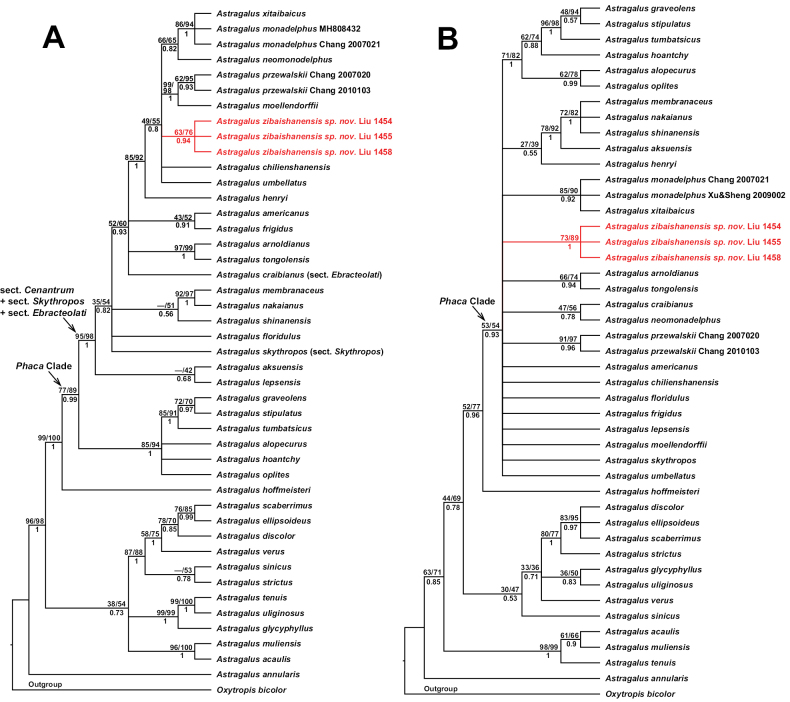
Bayesian 50% majority-rule consensus trees based on the nuclear ITS sequence **A.** and the combined plastid *psbA-trnH* and *trnL-F* sequences **B.** The Bayesian posterior probabilities (PP) are below the branches, the maximum parsimony and the maximum likelihood bootstrap support percentages (BSMP, BSML) are above the branches. A dash (–) indicates a branch that is not found in the maximum parsimony tree.

In the nuclear tree (Fig. 1A), three accessions of the new species clustered together (PP = 0.94, BSMP = 63%, BSML = 76%). The new species and *A. henryi* Oliv., *A. umbellatus* Bunge, *A. chilienshanensis* Y. C. Ho, *A. moellendorffii* Bunge, *A. przewalskii* Bunge, *A. monadelphus*, *A. xitaibaicus* and *A. neomonodelphus* formed a well-supported clade (PP = 1, BSMP = 85%, BSML = 92%). Within this clade, *A. monadelphus*, *A. xitaibaicus* and *A. neomonodelphus* clustered together (PP = 0.82, BSMP = 66, BSML = 65). *Astragalus skythropos* (A. sect. Skythropos) and *A. craibianus* (A. sect. Ebracteolati) nested with species of A. sect. Cenantrum, and they formed a well-supported clade (PP = 1, BSMP = 95%, BSML = 98%). The *Phaca* clade was well-supported (PP = 0.99, BSMP = 77%, BSML = 89%).

### ﻿Plastid data

In the plastid tree (Fig. 1B), three accessions of the new species clustered together (PP = 1, BSMP = 73%, BSML = 89%). *Astragalus monadelphus* and *A. xitaibaicus* formed a clade (PP = 0.92, BSMP = 85%, BSML = 90%). The *Phaca* clade was also supported (PP = 0.93, BSMP = 53%, BSML = 54%), but the relationships among species of the *Phaca* clade were largely unresolved in a polytomy.

### ﻿Taxonomy

#### ﻿ Astragalus zibaishanensis

Taxon classificationPlantaeFabalesFabaceae

P.L. Liu & L.L. Xun, sp. nov. (A. sect. Cenantrum Bunge)

74EA866E-7C02-5E96-8E6E-6C707FDBB0BA

urn:lsid:ipni.org:names:77372165-1

Figs 2, 3

##### Type.

**China** • Shaanxi Province, Fengxian County, north slope of Mt. Zibaishan, 33°41'49.6"N, 106°45'21.3"E, on stony slope in grasses, 2311 m above sea level (a. s. l.), 13 June 2023, *P. L. Liu 1458* (Holotype, WUK!; Isotypes, WUK!, WNU!).

**Figure 2.**
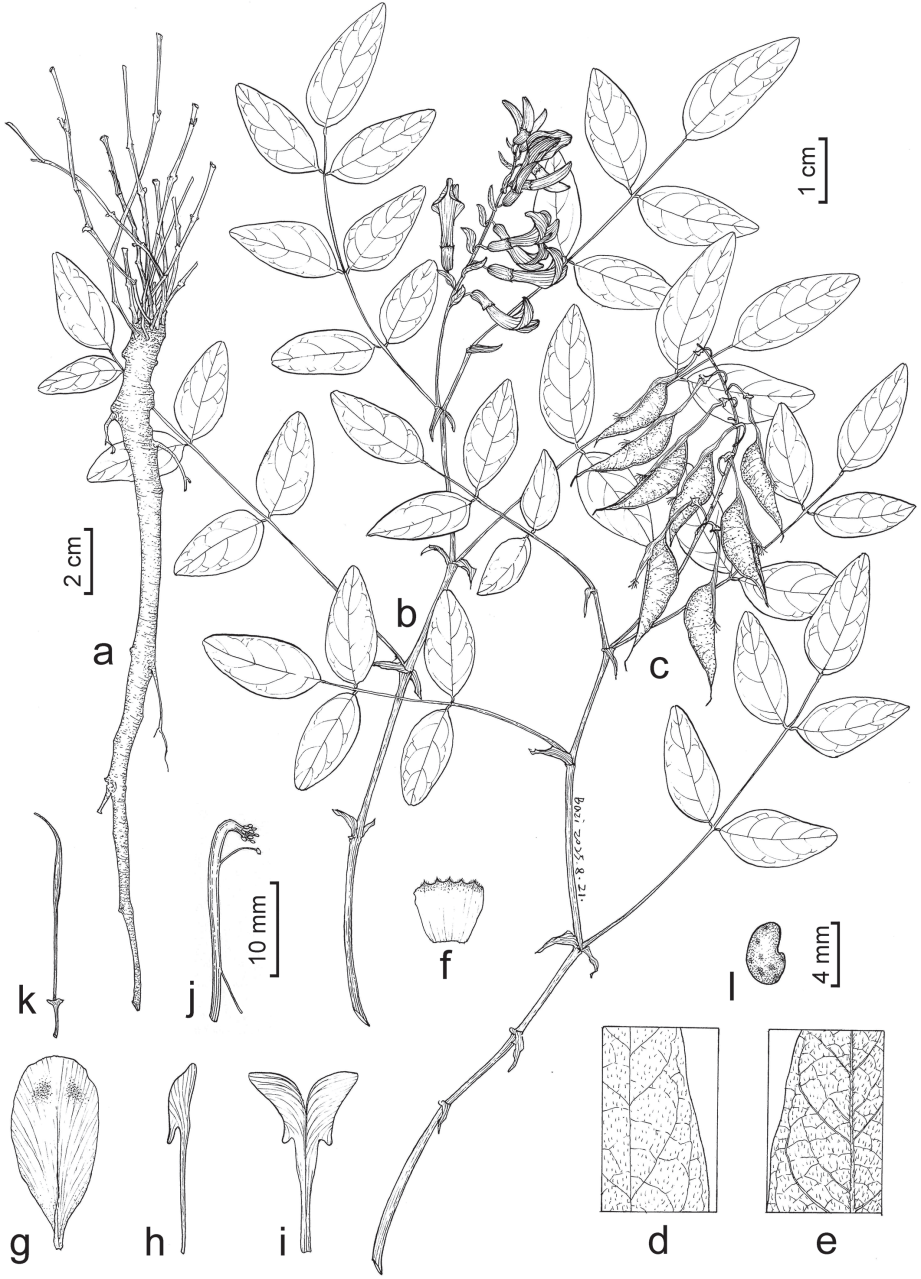
Illustration of *Astragalus zibaishanensis*. **a.** Root with basal stems; **b.** Upper part of a flowering plant; **c.** Upper part of a fruiting plant; **d.** Leaflet, adaxial view; **e.** Leaflet, abaxial view; **f.** Split calyx, inner side; **g.** Standard; **h.** Wing; **i.** Keels; **j.** Androecium; **k.** Pistil; **l.** Seed. Drawn by Yi-Fan Li.

##### Diagnosis.

This new species is similar to *A. monadelphus*, *A. xitaibaicus* and *A. neomonodelphus* by having yellow petals, pseudomonadelphous androecium, and stipitate legumes, but can be distinguished by its stem 0.9–1.5 mm in diameter; leaflets 5–7, hairy adaxially and abaxially; calyx teeth triangular, ca. 0.5 mm long; standard 17–20 mm long; wings 18–21 mm long; keels 17–20 mm long; pistil glabrous; legume glabrous, with a stipe 10–11 mm long (Table 1).

**Table 1.** Morphological comparison of *Astragalus zibaishanensis*, *A. monadelphus*, *A. xitaibaicus* and *A. neomonodelphus*.

	*A. zibaishanensis*	*A. monadelphus*	*A. xitaibaicus*	*A. neomonodelphus*
Stem diameter	0.9–1.5 mm	2–4 mm	2–4 mm	2–3 mm
Leaflets	5–7; hairy adaxially and abaxially	9–17; glabrous adaxially, hairy abaxially	11–15; glabrous adaxially, glabrous or hairy abaxially	9–15; glabrous adaxially and abaxially
Calyx teeth	triangular, ca. 0.5 mm long	subulate, 2.0–4.0 mm long	subulate, 2.5–3.0 mm long	two adaxial teeth triangular, 1.0–1.5 mm long, three abaxial teeth narrowly triangular, 1.5–3.0 mm long
Standard	17–20 mm long	10–13 mm long	ca. 11 mm long	16–17 mm long
Wings	18–21 mm long	10–12 mm long	ca. 11 mm long	15–16 mm long
Keels	17–20 mm long	10–11 mm long	ca. 10 mm long	15–16 mm long
Pistil	glabrous	hairy	hairy	hairy
Legume	glabrous; stipe 10–11 mm long	hairy; stipe 5–6 mm long	hairy; stipe 5–6 mm long	hairy; stipe 7–8 mm long

**Figure 3.**
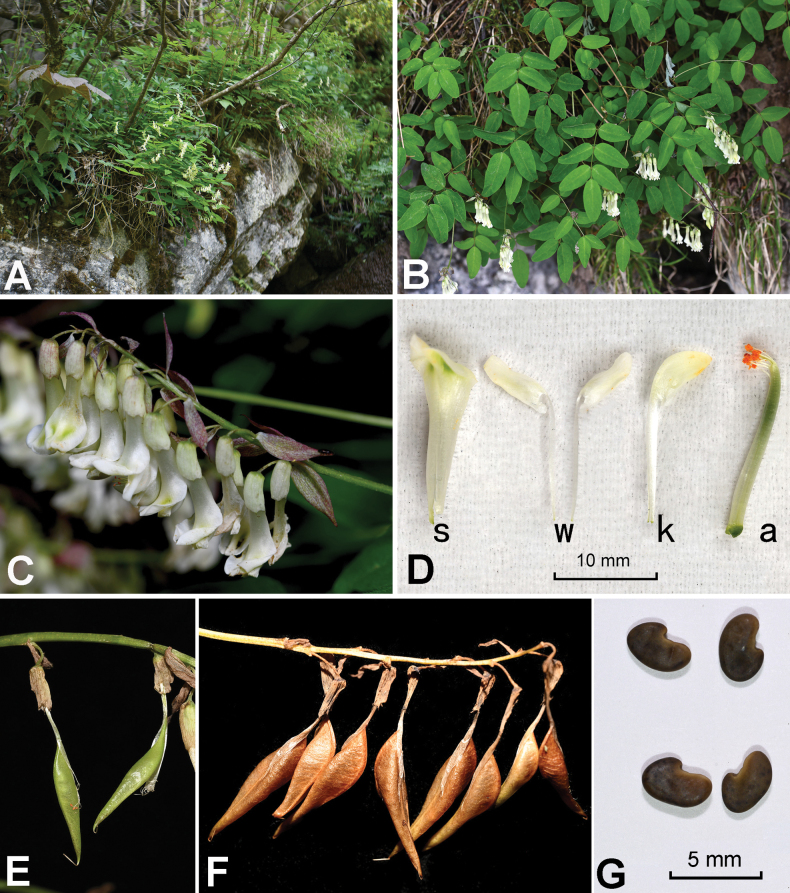
Photos of *Astragalus zibaishanensis* from the field. **A.** Habitat; **B.** Upper part of a flowering plant; **C.** Raceme; **D.** A dissected flower (s, standard; w, wings; k, keels; a, androecium); **E.** Young legumes; **F.** Mature legumes; **G.** Seeds. Photoed by Lu-Lu Xun.

##### Description.

Perennial herbs, 20–30 cm tall; main root 6–10 mm in diameter. Stems cespitose, ascending, unbranched, slightly zigzag, slender, 0.9–1.5 mm in diameter; internodes sparsely pubescent or glabrous. Leaves imparipinnate, alternate, 7–11 cm long; stipules free, often reflexed, lanceolate or ovate, 6–9 × 1–3 mm, brown or green, glabrous; rachises sparsely pubescent or glabrous; leaflets 5–7, opposite; petiolules 1.0–1.5 mm long, pubescent; leaflet blades ovate or oblong, 15–30 × 7–15 mm, with white appressed simple hairs adaxially and abaxially, base wide cuneate or rounded, apex retuse or obtuse. Peduncles axillary, 3–8 cm long, sparsely pubescent or glabrous; racemes with 9–15 flowers; pedicels 4–5 mm long, sparsely pubescent; bracts lanceolate, 7–9 × 1.5–2.5 mm, glabrous. Calyx 6–7 mm long, inner side of calyx teeth with brown hairs, other parts of calyx glabrous; calyx teeth 5, triangular, equal, ca. 0.5 mm long. Petals light yellow; standard obovate, 17–20 × 7–8 mm, apex retuse or rounded, base attenuate; wings 18–21 × 1.8–2.0 mm, auricle narrowly triangular, ca. 1 mm long, claw 11–14 mm long; keels 17–20 × 2.5–3.0 mm, auricle triangular, ca. 0.8 mm long; androecium pseudomonadelphous, 18–21 mm long, the vexillary stamen (the single stamen which is close to the standard) is attached to other nine fused filaments at middle, and free from the fused filaments at base and apex; pistil 17–21 mm long, glabrous, stipe 7–10 mm long, style 4–5 mm long. Legume glabrous, with a stipe 10–11 mm long, inflated, unilocular, outline lanceolate, 18–22 mm long (excluding stipe), 5–6 mm wide. Seeds black-brown, 3.5–4.0 × 2.0–2.5 mm.

##### Phenology.

Flowering in May and June; fruiting in June and July; seeds mature in July.

##### Distribution and habitat.

**Figure 4.**
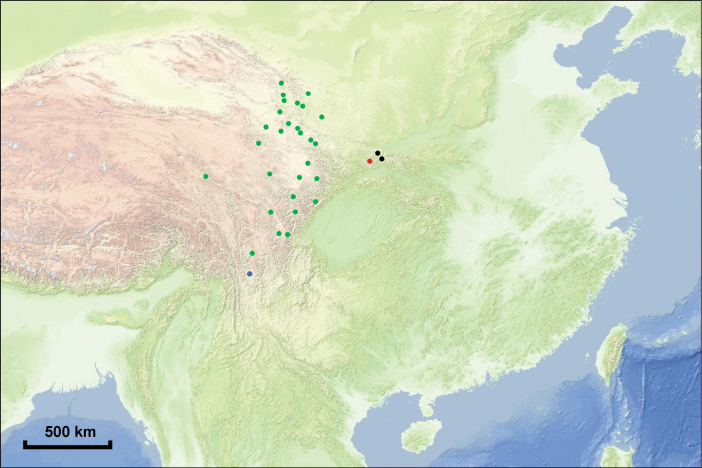
Distributions of *Astragalus zibaishanensis* (red dot), *A. monadelphus* (green dots), *A. xitaibaicus* (black dots) and *A. neomonodelphus* (blue dot). Maps from map.tianditu.gov.cn

*Astragalus zibaishanensis* is distributed on the north slope of Mt. Zibaishan in Fengxian County, Shaanxi, China (Fig. 4). It grows on stony slope in forest, 2300–2400 m a. s. l.

##### Etymology.

The epithet *zibaishanensis* derives from the type locality of the new species, Mt. Zibaishan (紫柏山). The Chinese vernacular name for this new species is 紫柏山黄耆 (zǐ bǎi shān huáng qí).

##### Other specimens checked (Paratypes).

**China** • Shaanxi Province, Fengxian County, north slope of Mt. Zibaishan, 33°41'47.0"N, 106°45'18.7"E, 2380 m a. s. l., 24 May 2022, *P. L. Liu 1454* (WUK!, WNU!); • same locality, 33°41'43.0"N, 106°45'10.3"E, 2391 m a. s. l., 24 May 2022, *P. L. Liu 1455* (WUK!); • same locality, 33°41'47.0"N, 106°45'18.3"E, 2385 m a. s. l., 16 July 2025, *P. L. Liu 1757* (WUK!).

##### Citations of comparative species.

***Astragalus monadelphus*** Bunge ex Maxim., Bull. Acad. Imp. Sci. Saint-Pétersbourg 24: 32. 1878. **Lectotype** (Podlech and Sytin 1996): West China, Prov. Kansu, vii 1872, *Prezewalski* (LE, sheet marked as lectotypus; drawing and fragments at K!); **isolectotypes**: LE, P, PE!.

***Astragalus xitaibaicus*** (K. T. Fu) Podlech & L. R. Xu, Novon 14 (2): 222. 2004. **Holotype**: Shaanxi, Xitaibai Shan, Dingpeng Shan, alt. 3300 m, in grassland on mountain ridge, 9 August 1957, *Kun-Tsun Fu 10321* (WUK!); **isotype**: WUK!.

***Astragalus neomonodelphus*** H. T. Tsai & T. T. Yu, Bull. Fan Mem. Inst. Biol. Bot. 9: 263. 1940. **Holotype**: Yunnan, Chungtien, Sianrentung, alt. 3400 m, grassy slope, herb, fl. yellow, 12 July 1937, *T. T. Yü 12120* (KUN); **isotypes**: PE!, HUH!.

## ﻿Discussion

*Astragalus zibaishanensis* is unique in *Astragalus* by having pseudomonadelphous androecium. The vexillary stamen is attached to other nine fused filaments in the middle, and free from the fused filaments at base and apex (Fig. 2j). In contrast, a monadelphous androecium is characterized by the completely joined filaments forming a tube of ten stamens (Rodríguez-Riaño et al. 1999). In Fabaceae, monadelphous androecium is found in tribe Genisteae, the genus *Ononis* L., and *Galega officinalis* L., while pseudomonadelphous androecium is found in the genera *Coronilla* L., *Lathyrus* L., *Medicago* L., *Vicia* L., *Pisum* L., *Trifolium* L., *Robinia* L. and others (Rodríguez-Riaño et al. 1999; Sinjushin et al. 2022). In *Astragalus*, most species have diadelphous androecium, and only *A. monadelphus*, *A. xitaibaicus*, *A. neomonodelphus* and *A. changmuicus* C. C. Ni & P. C. Li had been recorded to have monadelphous androecium (Li and Ni 1979; Xu and Podlech 2010; Podlech and Zarre 2013). However, careful observation showed that *A. monadelphus* has pseudomonadelphous androecium (Liu et al. 2010), which is similar to that of *A. zibaishanensis*. Considering the close phylogenetic relationship of *A. monadelphus*, *A. xitaibaicus* and *A. neomonodelphus* (Fig. 1A), further observations of the androecium type of *A. xitaibaicus* and *A. neomonodelphus* are needed.

**Figure 5.**
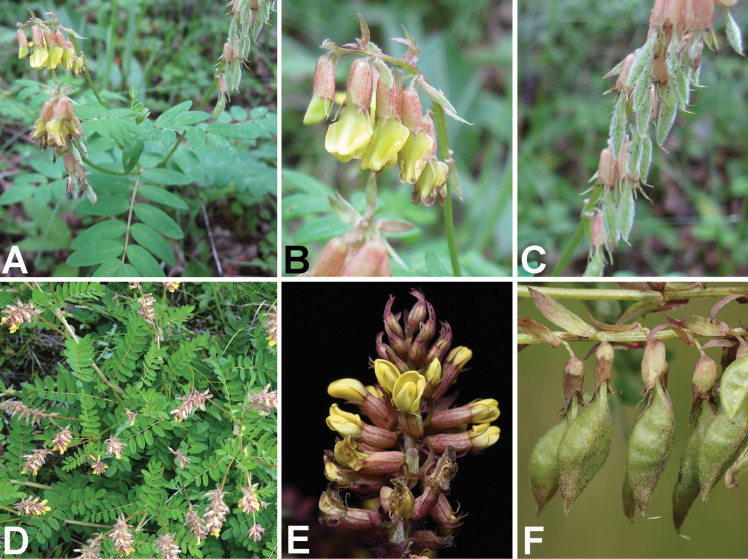
Photos of *Astragalus monadelphus* (**A–C**) and *A. xitaibaicus* (**D–F**) from the field. **A, D.** Upper part of a flowering plant; **B, E.** Raceme; **C, F.** Legumes. **A–C** photoed by Lang-Ran Xu; **D** photoed by Lu-Lu Xun; **E** photoed by Yuan Lu; **F** photoed by Jian-Quan Tang.

In morphology, *A. zibaishanensis* can be distinguished from *A. monadelphus*, *A. xitaibaicus* (Fig. 5) and *A. neomonodelphus* by the characters listed in Table 1. Besides, these four species are allopatric in geographic distribution. *Astragalus monadelphus* is widely distributed in Gansu, Qinghai and Sichuan Provinces; *A. neomonodelphus* is found in northwestern Yunnan Province; *A. xitaibaicus* is found in Taibai and Yangxian Counties in Shaanxi Province; *A. zibaishanensis* is only known from Mt. Zibaishan in Shaanxi Province at present (Fig. 4).

In the monograph of Old World *Astragalus*, Podlech and Zarre (2013) offered a practical key to 41 species in A. sect. Cenantrum (pages 171–174). We could insert the 42^nd^ species, *A. zibaishanensis*, to the key, and modify the key as follows (begins with the original numbering).

### ﻿Modified key to the species of Astragalus sect. Cenantrum in Old World

**Table:** 

4	Leaflets 3–5; inflorescence paniculate	***A. henryi***
–	Leaflets at least 5; inflorescence racemose	**5**
5	Leaflets (11–) 13–25; bracts 2–6 mm	**6 (original contents, omitted)**
–	Leaflets 5–13; bracts 5–20 mm	**10**
10	Leaflets hairy adaxially and abaxially; androecium pseudomonadelphous	***A. zibaishanensis***
–	Leaflets glabrous adaxially, hairy abaxially; androecium diadelphous	**species in the original key numbering 10 to 13 (omitted)**

## Supplementary Material

XML Treatment for Astragalus zibaishanensis

## ﻿Additional information

### Use of AI

No use of AI was reported.

### Author ORCIDs

Pei-Liang Liu https://orcid.org/0000-0001-6566-7824

Lu-Lu Xun https://orcid.org/0000-0002-6861-5883

Yuan Lu https://orcid.org/0000-0002-0329-0803

Bin Li https://orcid.org/0000-0001-9254-8003

Ming Yue https://orcid.org/0000-0002-8618-1126

### Data availability

The newly generated DNA sequences have been deposited in GenBase and the accession numbers can be found in Appendix 1.

## ﻿Appendix 1

Taxon name, geographical locality, voucher, herbarium code, and GenBase (https://ngdc.cncb.ac.cn/genbase) accession number for the newly generated sequences (indicated by an asterisk *). For each sample, accession numbers are given for the ITS, *psbA-trnH*, *trnL-F* sequences. A dash (–) indicates a missing sequence. Sequences acquired from the GenBank (https://www.ncbi.nlm.nih.gov/) are listed with taxon names and accession numbers.

*Astragalus acaulis* Baker: SAMN15406160;

*Astragalus aksuensis* Bunge: SAMN15406183;

*Astragalus alopecurus* Pallas: SAMN15406163 ;

*Astragalus americanus* (Hook.) M. E. Jones: SAMN15406142;

*Astragalus annularis* Forssk.: SAMN15406164 ;

*Astragalus arnoldianus* N. D. Simpson: China, Sichuan, Luding, *P. L. Liu 1355* (WNU), C_AA122198*, C_AA121464*, C_AA121450*;

*Astragalus chilienshanensis* Y. C. Ho: China, Qinghai, Ledu, *L. R. Xu & G. W Sheng 2009-003* (WUK), C_AA122199*, C_AA121465*, C_AA121451*;

*Astragalus craibianus* N. D. Simpson: SAMN15406173;

*Astragalus discolor* Bunge: SAMN15406200;

*Astragalus ellipsoideus* Ledeb.: SAMN15406192;

*Astragalus floridulus* Podlech: MZ198526, NC_083391;

*Astragalus frigidus* (L.) A. Gray: China, Shanxi, Wuzhai, *P. L. Liu 1462-1* (WNU), C_AA122200*, C_AA121466*, C_AA121452*;

*Astragalus glycyphyllus* L.: SAMN15406130;

*Astragalus graveolens* Buch.-Ham. ex Benth.: SAMN15406162;

*Astragalus henryi* Oliv.: SAMN15406197;

*Astragalus hoantchy* Franch.: SAMN15406159;

*Astragalus hoffmeisteri* (Klotzsch) Ali: SAMN15406172;

*Astragalus lepsensis* Bunge: China, Xinjiang, Gongnaisi, *L. R. Xu 1435* (WUK), –, C_AA121467*, C_AA121453*;

*Astragalus lepsensis* Bunge: AF359752;

*Astragalus membranaceus* Bunge: China, Shaanxi, Chang’an, *B. Li LB24406* (XBGH), C_AA122201*, C_AA121468*, C_AA121454*;

*Astragalus moellendorffii* Bunge: China, Ningxia, Liupanshan, *L. R. Xu 96-204* (WUK), C_AA122202*, C_AA121469*, C_AA121455*;

*Astragalus monadelphus* Bunge ex Maxim.: China, Qinghai, Ledu, *L. R. Xu & G. W Sheng 2009-002* (WUK), –, C_AA121470*, –;

*Astragalus monadelphus* Bunge ex Maxim.: China, Gansu, Yuzhong, *Z. Y. Chang et al. 2007021* (WUK), C_AA122203*, C_AA121471*, C_AA121456*;

*Astragalus monadelphus* Bunge ex Maxim., MH808432;

*Astragalus muliensis* Hand.-Mazz.: MZ198530, NC_083909;

*Astragalus nakaianus* Y. N. Lee: KC262198, NC_028171;

*Astragalus neomonodelphus* H. T. Tsai & T. T. Yu, SAMN15406153;

*Astragalus oplites* Benth. ex R. Parker: SAMN15406189;

*Astragalus przewalskii* Bunge: China, Qinghai, Xunhua, *Z. Y. Chang et al. 2010103* (WUK), C_AA122205*, C_AA121473*, C_AA121458*;

*Astragalus przewalskii* Bunge: China, Gansu, Yuzhong, *Z. Y. Chang et al. 2007020* (WUK), C_AA122204*, C_AA121472*, C_AA121457*;

*Astragalus scaberrimus* Bunge: SAMN15406155;

*Astragalus shinanensis* Ohwi: LC760273, LC760283, –;

*Astragalus sinicus* L.: SAMN15406198;

*Astragalus skythropos* Bunge: SAMN15406208;

*Astragalus stipulatus* D. Don: MZ198531, NC_083911;

*Astragalus strictus* Graham ex Benth.: SAMN15406213;

*Astragalus tenuis* Turcz.: SAMN15406203;

*Astragalus tongolensis* Ulbr.: China, Xizang, Jiacha, *P. L. Liu 1531* (WNU), C_AA122206*, C_AA121474*, C_AA121459*;

*Astragalus tumbatsicus* C. Marquand & Airy Shaw: MZ198541, NC_083912;

*Astragalus uliginosus* L.: SAMN15406204;

*Astragalus umbellatus* Bunge: AF121683 &amp; MG236337, –, AF126988;

*Astragalus verus* Oliv.: SAMN15406193;

*Astragalus xitaibaicus* (K. T. Fu) Podlech & L. R. Xu: China, Shaanxi, Yangxian, *P. L. Liu 1651* (WNU), C_AA122207*, C_AA121475*, C_AA121460*;

*Astragalus zibaishanensis* P. L. Liu & L. L. Xun: China, Shaanxi, Fengxian, *P. L. Liu 1454* (WNU), C_AA122208*, C_AA121476*, C_AA121461*;

*Astragalus zibaishanensis* P. L. Liu & L. L. Xun: China, Shaanxi, Fengxian, *P. L. Liu 1455* (WNU), C_AA122209*, C_AA121477*, C_AA121462*;

*Astragalus zibaishanensis* P. L. Liu & L. L. Xun: China, Shaanxi, Fengxian, *P. L. Liu 1458* (WNU), C_AA122210*, C_AA121478*, C_AA121463*;

*Oxytropis bicolor* Bunge: SAMN15406215.
